# Baseline hematuria in autosomal dominant polycystic kidney disease

**DOI:** 10.1093/ckj/sfag300

**Published:** 2026-08-31

**Authors:** Andreas Serra, Jana Henschkowski Serra, Maximilian Severin, Stefan Russmann

**Affiliations:** Suisse ADPKD, Klinik Hirslanden, Zürich, Switzerland; Epidemiology, Biostatistics and Prevention Institute (EBPI), University of Zürich, Switzerland; Suisse ADPKD, Klinik Hirslanden, Zürich, Switzerland; Suisse ADPKD, Klinik Hirslanden, Zürich, Switzerland; Suisse ADPKD, Klinik Hirslanden, Zürich, Switzerland

**Keywords:** ADPKD, disease progression, eGFR slope, hematuria, kidney survival

## Abstract

**Background:**

Hematuria is common in autosomal dominant polycystic kidney disease (ADPKD), but whether baseline hematuria predicts long-term kidney outcomes is unknown. We tested its association with kidney function decline, renal survival, and volume growth.

**Methods:**

In a single-center ADPKD cohort, 175 patients had standardized urinary sediment assessment in a baseline period (2006–11); 173 (99%) were linked to a registry (follow-up up to 20 years). Microscopic hematuria was >5 erythrocytes per high-power field; macroscopic was from clinical history. Outcomes were the estimated glomerular filtration rate (eGFR) slope, a composite of end-stage kidney disease or eGFR halving, and total kidney volume (TKV) growth, adjusted for baseline eGFR, age, and sex.

**Results:**

Baseline hematuria was present in 45 patients (26%; microscopic 15%, macroscopic 17%) and was associated with faster adjusted eGFR decline [mixed-model differential slope: any −1.46 ml/min/1.73 m²/year (95% confidence interval {CI}, −2.12 to −0.80); macroscopic −1.73 (−2.53 to −0.93); microscopic −1.53 (−2.34 to −0.73); *P *< .001 for all]. The composite end point was more frequent with hematuria (15-year event-free survival 33% vs. 58%; *P *= .002); macroscopic hematuria was independently associated [adjusted hazard ratio 1.97 (1.08–3.58); *P *= .03]. Hematuria was not associated with TKV growth (all *P *> .3). Associations persisted after adjustment for baseline kidney volume and albuminuria and in never-treated patients, were dose-dependent for macroscopic hematuria, and strengthened for microscopic hematuria when infection-associated episodes were excluded.

**Conclusions:**

Baseline hematuria—particularly macroscopic and non–infection-associated microscopic—independently predicts accelerated functional decline and worse renal survival. Because this prognostic signal is functional rather than volume-driven, this simple, routinely available finding may complement imaging-based risk markers.

KEY LEARNING POINTS **What was known:**

- Hematuria is common in ADPKD, but most evidence linking it to outcomes comes from cross-sectional studies or analyses of gross hematuria with limited follow-up.
- Total kidney volume is the best-validated structural predictor of progression, yet requires standardized volumetric imaging that is not universally available.
- Whether hematuria documented early in the disease course predicts the subsequent trajectory of kidney function had not been established in long-term follow-up.

**This study adds:**

- In a single-center ADPKD cohort with follow-up up to 20 years, baseline hematuria independently predicted faster eGFR decline and worse renal survival, robust to adjustment for baseline kidney volume, albuminuria, and tolvaptan treatment.
- The prognostic signal was specific to functional decline: hematuria was not associated with the rate of kidney volume growth.
- The association was dose-dependent for macroscopic hematuria and strengthened for microscopic hematuria when infection-associated episodes were excluded.

**Potential impact:**

- Hematuria, a simple and routinely available finding, may aid identification of patients at higher risk of progression.
- It could complement, rather than duplicate, imaging-based risk stratification, particularly where volumetric imaging is unavailable.
- Careful definition of hematuria—distinguishing intrinsic from infection-related episodes—may improve its prognostic value.

## INTRODUCTION

Autosomal dominant polycystic kidney disease (ADPKD) is the most common inherited kidney disorder and a leading cause of kidney failure [1]. Its course is highly variable: some patients reach kidney failure in mid-adulthood, whereas others retain adequate function into old age [2, 3]. Reliable identification of patients at high risk of rapid progression is therefore central to clinical management, particularly now that disease-modifying therapy is available and benefits most those with rapidly progressive disease [4, 5].

Total kidney volume (TKV), formalized in the Mayo imaging classification, is the best-validated structural predictor of progression [6–8] and, together with age, estimated glomerular filtration rate (eGFR), sex, and genotype, underpins current risk-stratification tools [9, 10]. However, imaging-based staging requires standardized volumetric measurement that is not universally available, and structural markers do not fully capture functional heterogeneity. Simple, inexpensive markers adding prognostic information would be valuable, especially where volumetric imaging is unavailable.

Hematuria is a frequent manifestation of ADPKD, arising from cyst hemorrhage, nephrolithiasis, infection [11]. Episodes of gross hematuria have long been associated with larger kidneys and more advanced disease, and early observations identified hematuria among the clinical features marking a more severe phenotype [12, 13]. Microscopic hematuria is even more common but has been studied mainly cross-sectionally, with its heterogeneous causes rarely distinguished [14]. Whether hematuria documented early in the disease course predicts the subsequent trajectory of kidney function—as opposed to merely reflecting concurrent disease severity—has not been established in long-term follow-up.

We therefore used a well-characterized ADPKD cohort with standardized baseline urinary sediment assessment, linked to a longitudinal registry with follow-up extending up to two decades, to test whether baseline microscopic and macroscopic hematuria independently predict the rate of kidney function decline, renal survival, and kidney volume growth.

## MATERIALS AND METHODS

### Study design and population

We conducted a longitudinal cohort study using the Suisse registry, a prospective observational cohort of patients with ADPKD. Patients diagnosed with ADPKD according to Ravine criteria [15, 16], aged 18–50 years and with an eGFR >60 ml/min/1.73 m², qualified for enrolment. Participants underwent clinical and laboratory examinations and magnetic resonance imaging (MRI) at baseline and at 6–12-monthly follow-up visits. For this analysis, the baseline (exposure) period comprised cohort visits in 2006–11, when standardized urinary sediment microscopy was systematically performed; the outcome period comprised all subsequent registry follow-up through 2026. Patients with at least one baseline-period visit including urinary sediment assessment were eligible. The study was conducted in accordance with the Declaration of Helsinki and approved by the cantonal ethics committee (KEK 1178); all participants provided written informed consent, including for long-term linkage of baseline data to the clinical registry.

### Assessment of hematuria

At each visit, urine was analyzed by reagent strip (Combur-10; Roche Diagnostics) with automated reading, followed by phase-contrast microscopy of the urinary sediment at ×400 magnification, performed according to European Urinalysis Guidelines [17]. Erythrocytes were quantified per high-power field (HPF) and recorded on an ordinal sediment scale. Microscopic hematuria was defined as a sediment erythrocyte rank ≥2, corresponding to >5 erythrocytes per HPF; a single visit meeting this threshold constituted one episode. Patients were classified as having any hematuria if they had at least one episode of microscopic or macroscopic hematuria during the baseline period; recurrent microscopic hematuria was defined as ≥2 qualifying episodes. Throughout, baseline hematuria denotes hematuria documented during the standardized baseline window (2006–11). Macroscopic hematuria was ascertained from the clinical record as a physician-documented episode of visible (gross) hematuria. Non-urinary sources (e.g. menstruation) and stone-related episodes (urolithiasis) were reviewed and not counted, and episodes coinciding with a documented or suspected urinary-tract or cyst infection were flagged, with their exclusion examined in sensitivity analyses (Supplementary Table S1); dedicated concurrent urine culture was not systematically available. Urinary erythrocyte morphology was assessed by phase-contrast microscopy, with erythrocyturia classified as glomerular when >20% of erythrocytes were dysmorphic [18].

### Registry linkage and longitudinal measurements

Baseline-period patients were linked to the longitudinal clinical registry by their unique patient identifier. Serum creatinine, kidney imaging, blood pressure and clinical status were extracted over the entire follow-up. Serum creatinine was measured with an isotope-dilution mass spectrometry-traceable assay, and eGFR was calculated using the 2021 race-free CKD-EPI creatinine equation, with age computed at each measurement date and sex as recorded [19]. Implausible creatinine values [<0.3 or >17.0 mg/dl (<25 or >1500 µmol/l)] were excluded, and multiple same-day values were summarized by their median. TKV was measured by manual segmentation volumetry on unenhanced T1-weighted MRI, as previously described, as the sum of right and left kidney volumes [20]. Height-adjusted TKV (htTKV) was TKV divided by height in meters (ml/m), using the median of available height measurements. Hypertension status (known/treated) was recorded at each visit, and end-stage kidney disease (ESKD) events (dialysis initiation or kidney transplantation) and dates were obtained from the registry status records.

### Outcomes

The primary outcome was the rate of eGFR decline (ml/min/1.73 m²/year). The principal secondary outcomes were TKV growth (% per year, log-linear); a composite kidney end point of ESKD or eGFR halving from baseline; ESKD alone; and incident hypertension among patients normotensive at baseline. In a sensitivity analysis, the composite end point required a sustained eGFR halving, defined as two consecutive values ≤50% of baseline.

### Statistical analysis

**Figure 1:**
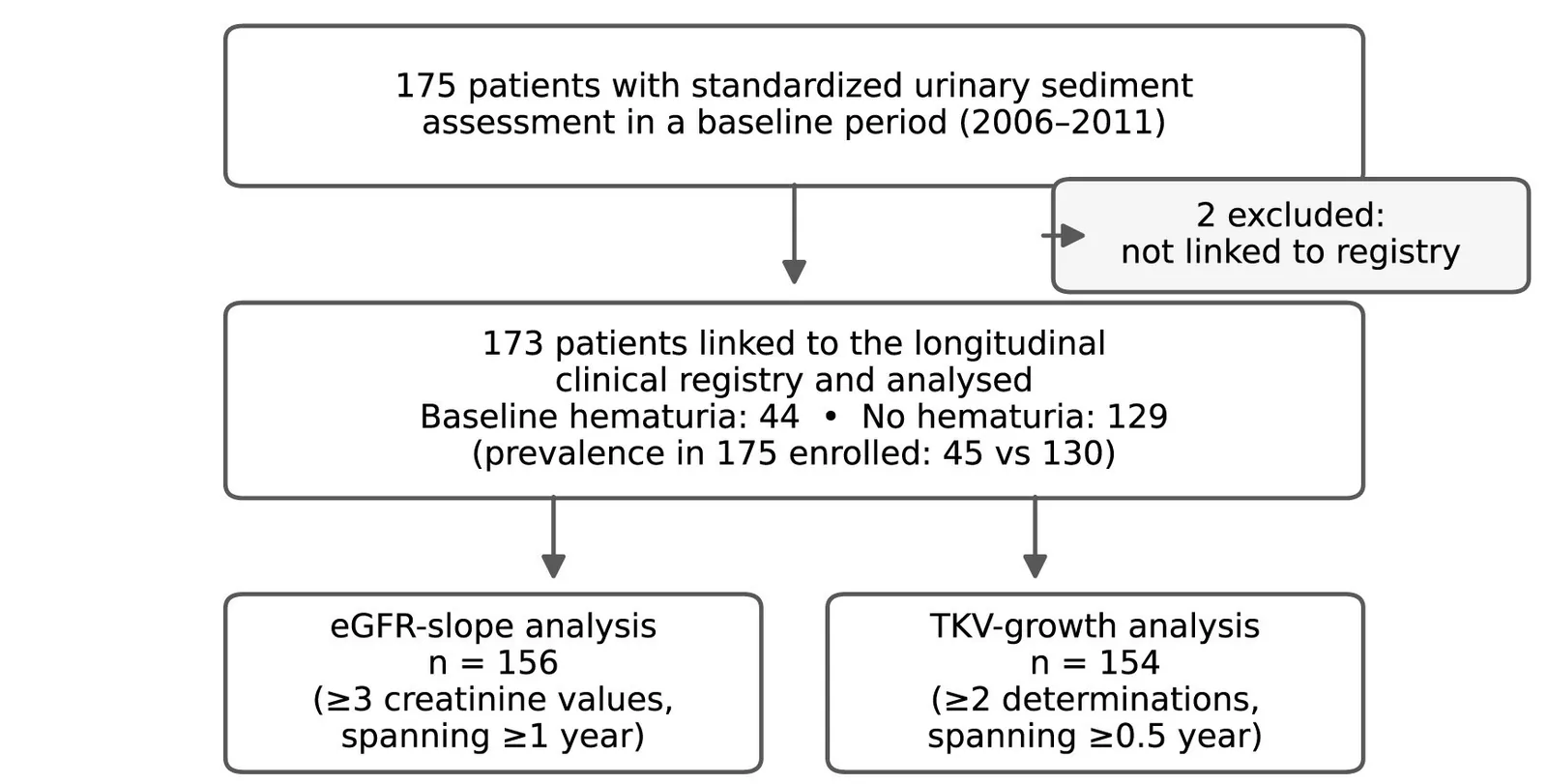
Participant flow and analytic samples. Of 175 enrolled patients, 173 were linked to the registry (44 with vs. 129 without baseline hematuria); analytic subsets differ by outcome (eGFR slope, *n* = 156; TKV growth, *n* = 154; composite end point and renal survival, *n* = 166).

Baseline characteristics were compared between hematuria groups using the Mann–Whitney *U* test for continuous variables and contingency tables for categorical variables. Individual eGFR slopes were estimated by ordinary least-squares regression of eGFR on time for patients with ≥3 measurements spanning ≥1 year, and TKV growth rates from patients with ≥2 determinations spanning ≥0.5 year. The association of hematuria with eGFR slope and TKV growth was assessed by linear regression adjusted for baseline eGFR, age, and sex. The study size reflected all eligible patients with baseline-period sediment assessment linked to the registry; no formal sample-size calculation was performed. Analyses used complete cases without imputation; the analytic sample size for each outcome is shown in Fig. 1.

As the principal analysis of functional decline, we fitted a linear mixed-effects model with random intercept and random slope for time, using all serial eGFR measurements; the interaction between time and hematuria status estimated the differential annual rate of eGFR decline, adjusted for age and sex. Time-to-event outcomes were analyzed by Kaplan–Meier estimation with the log-rank test and by Cox proportional-hazards regression adjusted for baseline eGFR, age, and sex; follow-up was measured from the baseline visit, and patients without an event were censored at their last creatinine measurement. Hematuria was modeled as any, macroscopic and microscopic hematuria in separate models.

In additional sensitivity analyses addressing potential confounding, the eGFR-slope and composite models were additionally adjusted for baseline TKV and height-adjusted TKV and, separately, for baseline albuminuria (log-transformed urinary albumin-to-creatinine ratio, UACR); the volume-adjusted estimate was also recomputed within the same subsample without the volume term to separate covariate from sample effects. Cumulative exposure was assessed by modeling macroscopic and microscopic hematuria as an ordinal episode count (0, 1, ≥2) with a linear trend test, including a microscopic variant excluding infection-associated episodes. TKV growth was re-estimated restricting to patients with determinations spanning at least 1 year, and more stringently at least three determinations over 1 year.

All analyses were performed in Python (statsmodels and lifelines). To assess robustness to informative censoring, a shared-parameter joint longitudinal–survival model (current-value parameterization, piecewise-constant baseline hazard) was additionally fitted in R using the JM package, [21] simultaneously modeling the eGFR trajectory (linear mixed-effects submodel) and time to ESKD (relative-risk submodel). Two-sided *P* values <.05 were considered statistically significant. Because some patients initiated disease-modifying therapy (tolvaptan or sirolimus) during follow-up, we performed sensitivity analyses censoring treated patients' eGFR series at therapy initiation and, alternatively, restricting to patients never treated with tolvaptan; we additionally modeled tolvaptan as a time-dependent covariate in the composite model and tested a hematuria-by-tolvaptan interaction. Because disease-modifying therapy is progression-indicated and lies on the causal pathway, it was not included as a baseline confounder in the primary models.

## RESULTS

### Study cohort

Of 175 patients enrolled in the Suisse ADPKD cohort with urinary sediment assessment during the baseline period (2006–11), 173 (98.9%) were successfully linked to the longitudinal clinical registry and included; two patients could not be linked and were excluded (Fig. 1). Participants contributed a median of 19–21 serum creatinine measurements and 11 TKV determinations each. A longitudinal eGFR slope could be estimated for 156 patients (≥3 creatinine values spanning ≥1 year) and a TKV growth rate for 154 patients (≥2 determinations spanning ≥0.5 year). Baseline characteristics stratified by baseline hematuria status are shown in Table 1. Patients with hematuria were older (median 35 vs. 30 years), had lower baseline eGFR (85 vs. 98 ml/min/1.73 m²) and larger baseline TKV (1130 vs. 737 ml), indicating more advanced disease at entry. They also had shorter observed follow-up (median 10.8 vs. 16.1 years), consistent with earlier kidney end points. Across the whole cohort, the median observed follow-up was 15.3 years [interquartile range (IQR), 5.1–18.9 years; maximum 20.2 years]. No baseline covariate was missing; differences in analytic sample size reflect prespecified longitudinal measurement requirements (Fig. 1), not missing data.

**Table 1:** Baseline characteristics by baseline hematuria status.

Characteristic	Hematuria (*n* = 44)	No hematuria (*n* = 129)
Age, years	35 (30–39)	30 (24–37)
Female sex, n (%)	16 (36)	57 (44)
Baseline eGFR, ml/min/1.73 m²	85 (72–94)	98 (83–112)
Baseline TKV, ml	1130 (630–1667)	737 (470–1094)
Baseline htTKV, ml/m	629 (401–903)	405 (269–633)
eGFR slope, ml/min/1.73 m²/year	−3.80 (−5.47, −2.46)	−2.14 (−3.46, −0.96)
TKV growth, %/year	6.5 (4.9–8.3)	5.8 (3.8–8.3)

eGFR, estimated glomerular filtration rate; htTKV, height-adjusted total kidney volume; IQR, interquartile range; TKV, total kidney volume.

Values are median (IQR) unless noted. Table values are based on the 173 registry-linked patients; of the 175 enrolled, 45 versus 130 had versus did not have hematuria, with one patient in each group not linked to the registry and therefore excluded from longitudinal analyses.

### Prevalence and types of baseline hematuria

During the baseline period, 45 of 175 patients (26%) had at least one episode of hematuria. Microscopic hematuria (sediment ≥rank 2, corresponding to >5 erythrocytes per HPF) occurred in 27 patients (15%), of whom 8 had recurrent episodes; macroscopic (gross) hematuria was documented in 29 patients (17%). Eleven patients had both. The remaining 130 patients had no hematuria and served as the reference group. Hematuria was ascertained at protocol-scheduled study visits and was asymptomatic; symptomatic urolithiasis was very rare and did not coincide with these assessments, and on phase-contrast microscopy erythrocytes were isomorphic, not meeting the definition of glomerular erythrocyturia.

### Baseline hematuria and eGFR decline

**Figure 2:**
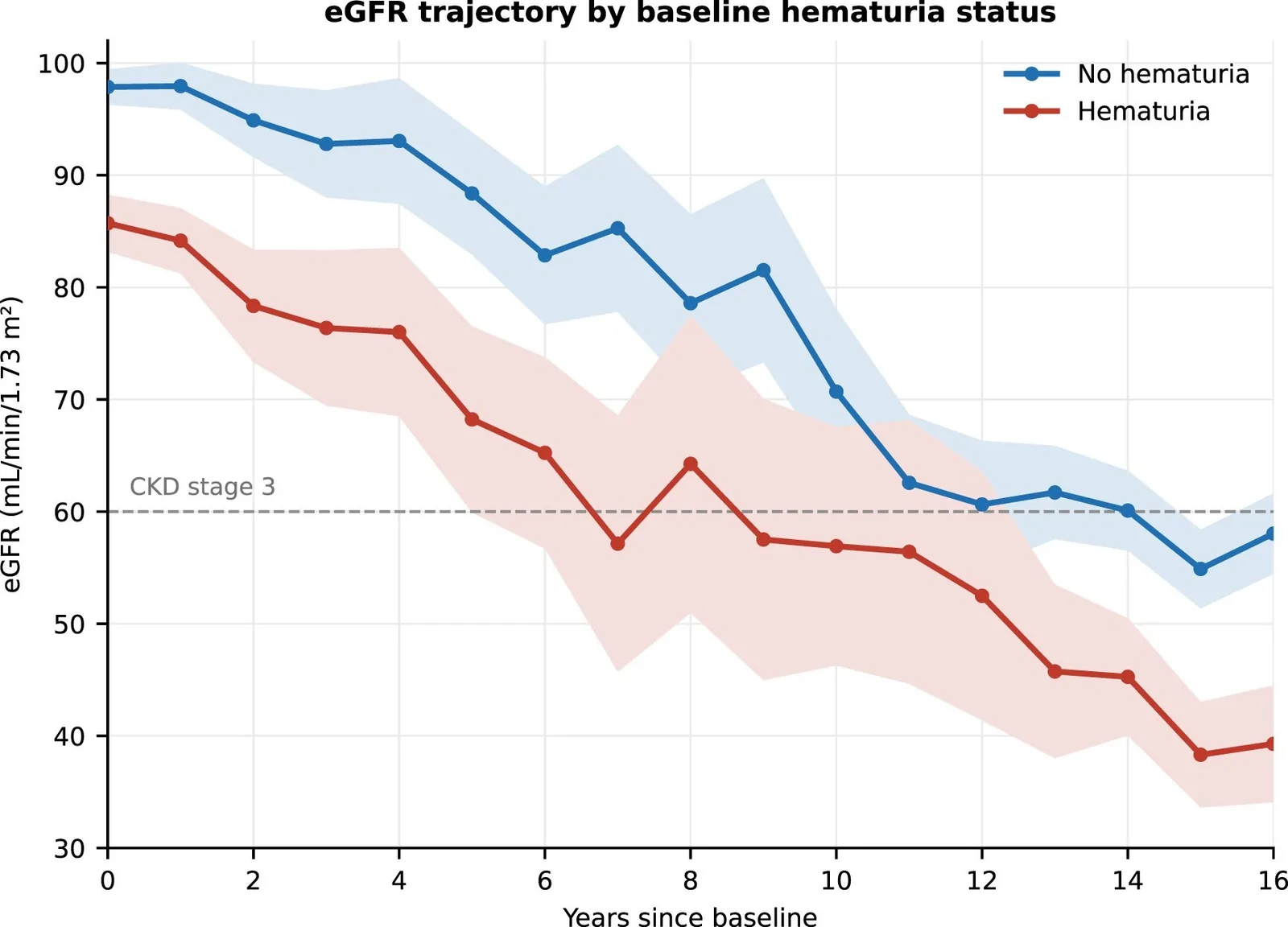
Mean eGFR trajectory by baseline hematuria status. Shaded bands denote standard error; the dashed line marks CKD stage 3 (eGFR 60 ml/min/1.73 m²).

The annual rate of eGFR decline was steeper with baseline hematuria (median −3.80 vs. −2.14 ml/min/1.73 m²/year; *P *< .001), for both macroscopic (−4.20 vs. −2.20; *P *< .001) and microscopic hematuria (−3.80 vs. −2.20; *P *= .004), and persisted after adjustment for baseline eGFR, age, and sex (Table 2). In linear regression of individual eGFR slopes, hematuria was associated with an additional decline of −1.44 ml/min/1.73 m²/year [95% confidence interval (CI), −2.34 to −0.54; *P *= .002]; the effect was strongest for macroscopic hematuria (−2.08; 95% CI, −3.12 to −1.04; *P *< .001). A linear mixed-effects model using all serial measurements confirmed and strengthened these findings: the differential annual eGFR decline was −1.46 (95% CI, −2.12 to −0.80) for any hematuria, −1.73 (95% CI, −2.53 to −0.93) for macroscopic and −1.53 (95% CI, −2.34 to −0.73) for microscopic hematuria (*P *< .001 for all three). Mean eGFR trajectories are shown in Fig. 2: the hematuria group started lower and declined more steeply, crossing the CKD stage 3 threshold (eGFR 60 ml/min/1.73 m²) several years earlier.

**Table 2:** Adjusted associations of baseline hematuria with progression outcomes.

Outcome (effect measure)	Any hematuria	Macrohematuria	Microhematuria
eGFR slope, OLS (β)	−1.44 (−2.34, −0.54); 0.002	−2.08 (−3.12, −1.04); <0.001	−1.03 (−2.13, 0.06); 0.06
eGFR slope, mixed model (β)	−1.46 (−2.12, −0.80); <0.001	−1.73 (−2.53, −0.93); <0.001	−1.53 (−2.34, −0.73); <0.001
TKV growth, %/year (β)	0.40 (−0.73, 1.52); 0.49	0.61 (−0.73, 1.95); 0.37	−0.24 (−1.56, 1.08); 0.72
ESKD or eGFR halving (HR)	1.58 (0.92, 2.71); 0.10	1.97 (1.08, 3.58); 0.03	1.51 (0.81, 2.80); 0.19
—sustained halving (HR)	1.54 (0.86, 2.75); 0.15	2.13 (1.14, 4.00); 0.02	1.28 (0.64, 2.56); 0.48
ESKD alone (HR)	1.33 (0.41, 4.34); 0.64	2.24 (0.67, 7.48); 0.19	0.55 (0.11, 2.64); 0.45
Incident hypertension (HR)	1.82 (0.86, 3.85); 0.12	2.28 (0.75, 6.96); 0.15	1.49 (0.57, 3.88); 0.42

CI, confidence interval; eGFR, estimated glomerular filtration rate; ESKD, end-stage kidney disease; OLS, ordinary least squares; TKV, total kidney volume.

Each cell: effect (95% CI); *P*. β in ml/min/1.73 m²/year or %/year; HR, hazard ratio. Linear (OLS slope and TKV growth), mixed-effects and Cox models were adjusted for baseline eGFR, age and sex; the incident-hypertension Cox model additionally included baseline TKV.

### Baseline hematuria and renal survival

**Figure 3:**
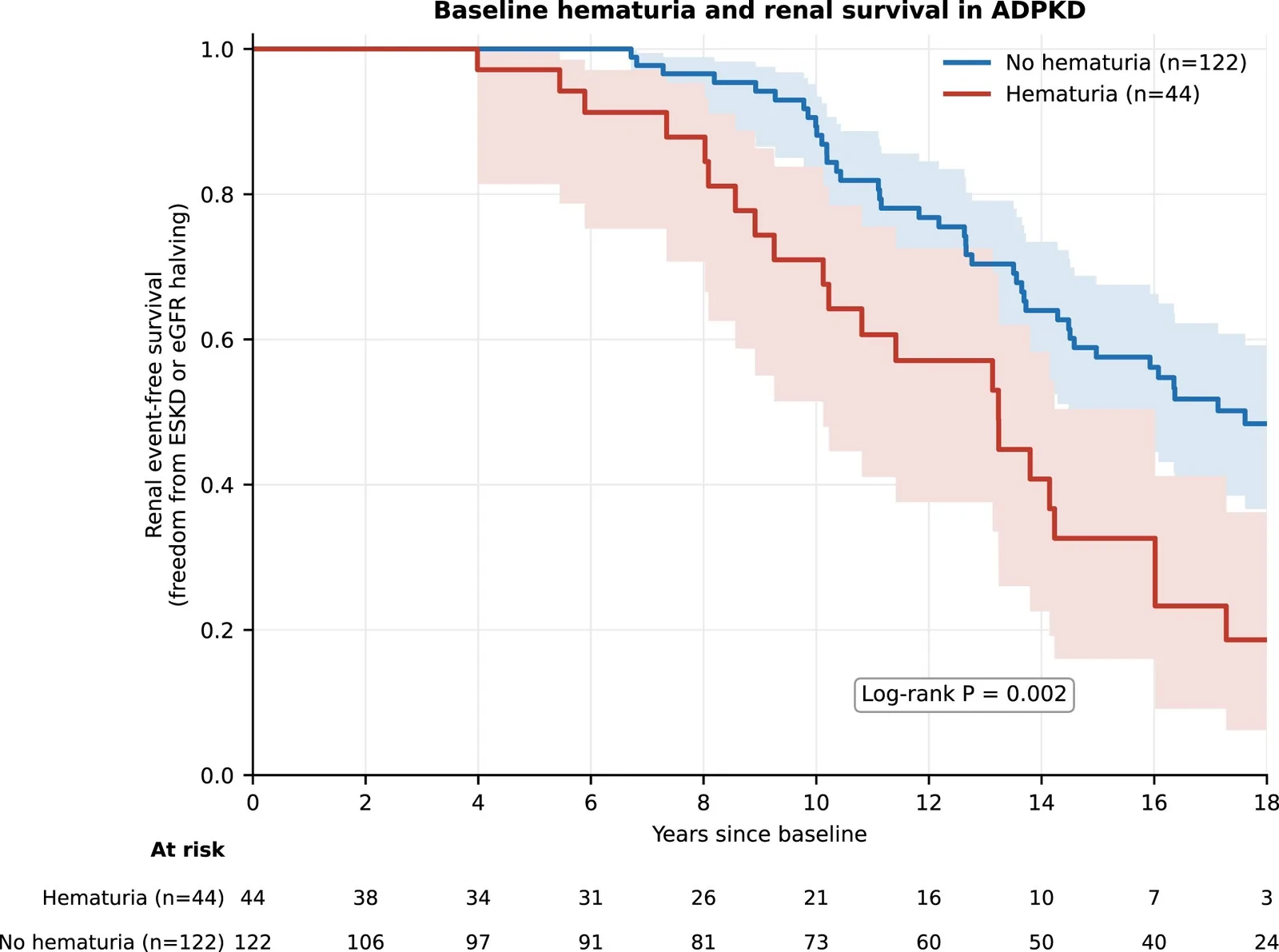
Renal event-free survival (freedom from ESKD or eGFR halving) by baseline hematuria status (Kaplan–Meier; log-rank *P *= .002). ESKD, end-stage kidney disease. Numbers at risk correspond to the composite-endpoint analytic set (*n* = 166: 44 with, 122 without hematuria).

**Figure 4:**
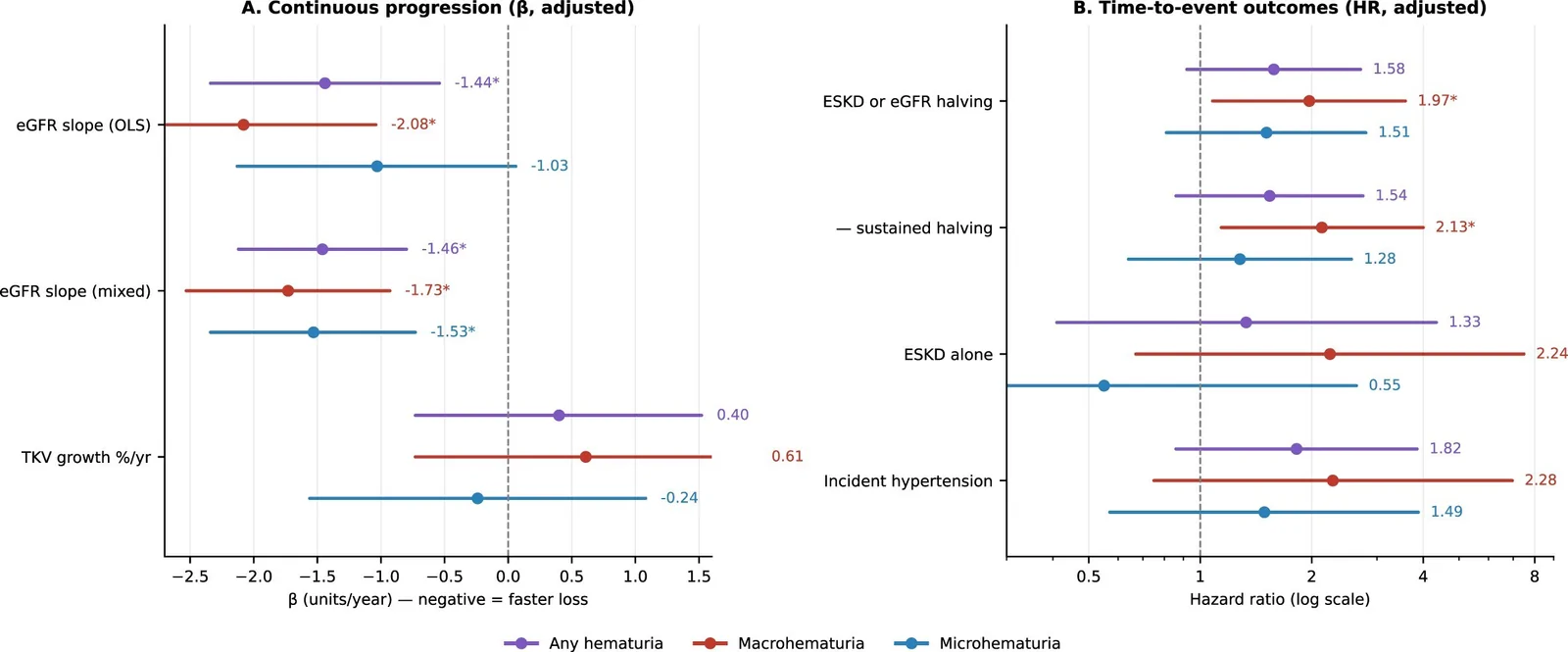
Forest plot of adjusted effect estimates across outcomes and hematuria definitions. Panel A: continuous outcomes (β); Panel B: time-to-event outcomes (hazard ratios). β, regression coefficient.

A composite kidney end point of ESKD (dialysis or kidney transplantation) or eGFR halving from baseline occurred in 64 patients. The 15-year cumulative incidence was markedly higher in patients with baseline hematuria (event-free survival 33% vs. 58%; log-rank *P *= .002; Fig. 3). In Cox models adjusted for baseline eGFR, age and sex, macroscopic hematuria was independently associated with the composite end point [hazard ratio (HR), 1.97; 95% CI, 1.08 to 3.58; *P *= .03], with consistent point estimates for any hematuria (HR 1.58; 95% CI, 0.92 to 2.71; *P *= .10) and microscopic hematuria (HR 1.51; 95% CI, 0.81 to 2.80; *P *= .19). Unadjusted associations were significant for all three exposures (any, HR 1.98, *P *= .009; macroscopic, HR 2.57, *P *= .001; microscopic, HR 1.95, *P *= .03). Adjusted estimates across all outcomes and definitions are summarized in Fig. 4.

### Baseline hematuria and kidney volume growth

In contrast to its association with functional decline, baseline hematuria was not associated with the rate of TKV growth, either unadjusted (median 6.5 vs. 5.8%/year; *P *= .20) or after adjustment (any hematuria, +0.40%/year, *P *= .49; macroscopic, +0.61%/year, *P *= .37; microscopic, −0.24%/year, *P *= .72; Table 2). This dissociation indicates that the prognostic signal of hematuria is specific to functional decline rather than reflecting a generally more aggressive, volume-driven phenotype.

### Secondary end points

For ESKD alone (13 events), point estimates were directionally consistent but did not reach significance given the limited number of events (macroscopic hematuria adjusted HR, 2.24; 95% CI, 0.67–7.48; *P *= .19); 15-year ESKD-free survival was 85% versus 94%. Among 58 patients normotensive at baseline, incident hypertension developed in 84%—in all 9 hematuria patients versus 39 of 48 without—with directionally consistent but non-significant hazard ratios (any adjusted HR 1.82, *P *= .12; macroscopic 2.28, *P *= .15); its near-universal development limited discriminatory value.

### Sensitivity analyses

Results were robust across alternative microhematuria definitions (Supplementary Table S1). Excluding episodes associated with urinary tract infection yielded the strongest association with eGFR decline (−1.28 ml/min/1.73 m²/year; *P *= .01), whereas the broadest definition including infection-associated episodes attenuated the estimate (−0.72; *P *= .13), suggesting that infection-related hematuria dilutes the prognostic signal. Restricting the composite end point to a sustained eGFR halving (confirmed by a subsequent measurement; 57 events) reinforced the association with macroscopic hematuria (adjusted HR, 2.13; 95% CI, 1.14 to 4.00; *P *= .02).

To address potential informative censoring—patients with hematuria had shorter observed follow-up—we performed a landmark analysis restricted to the first 8 years, during which censoring is minimal. The association of hematuria with eGFR decline was at least as strong within this window (any hematuria −1.60 [95% CI, −2.70 to −0.49], *P *= .005; macroscopic −2.49 [−3.76 to −1.21], *P *< .001), arguing for a genuine effect rather than a censoring artefact. Modeling time with a natural cubic spline did not improve fit over a linear term (Akaike information criterion 32 344 vs. 32 341), supporting the adequacy of the linear eGFR-slope model over the observed follow-up.

In the joint longitudinal–survival model, the differential eGFR slope for macroscopic hematuria was confirmed (−2.13 ml/min/1.73 m²/year; *P *< .001), with a significant association between the current eGFR level and the ESKD hazard (association parameter −0.36; *P *= .004). After accounting for the eGFR trajectory, the direct effect of macroscopic hematuria on ESKD was attenuated to the null (*P *= .76), indicating that its effect on kidney failure is mediated through accelerated functional decline. Because the differential slope was unchanged when the trajectory and event processes were modeled jointly, informative censoring is unlikely to explain the association.

Adjusting for baseline kidney volume did not abolish the association between macroscopic hematuria and eGFR decline: the differential slope was −1.68 ml/min/1.73 m²/year after adding baseline TKV (95% CI, −2.69 to −0.66; *P *= .001) and −1.65 after adding htTKV (−2.66 to −0.64; *P *= .002), versus −2.08 in the primary model; the estimate in the volume-adjusted subsample without the volume term was unchanged (−2.07), confirming a covariate rather than a sample effect. The composite end point was retained or strengthened [macroscopic hematuria HR 2.18 (1.19–4.00) with TKV, 2.16 (1.18–3.96) with htTKV; any hematuria became significant, HR 1.80 (1.04–3.11), *P *= .036]. Adjustment for baseline albuminuria (UACR available in 173 of 174 linked patients) had a similarly small effect on the macroscopic-hematuria slope [−1.96 (−3.02 to −0.91), *P *< .001; −1.68 with TKV added, *P *= .001] and composite association [HR 1.92 (1.04–3.54), *P *= .037]. Microscopic estimates were directionally consistent but attenuated (Table 3).

**Table 3:** Sensitivity analyses for the association of macroscopic baseline hematuria with eGFR decline and the composite kidney end point.

Model/analysis (macroscopic hematuria vs. none)	eGFR slope β (95% CI); *P*	Composite HR (95% CI); *P*
Primary model (eGFR, age, sex)	−2.08 (−3.12, −1.04); <0.001	1.97 (1.08–3.58); 0.03
+ baseline TKV	−1.68 (−2.69, −0.66); 0.001	2.18 (1.19–4.00); 0.01
+ baseline htTKV	−1.65 (−2.66, −0.64); 0.002	2.16 (1.18–3.96); 0.01
+ baseline albuminuria (UACR)	−1.96 (−3.02, −0.91); <0.001	1.92 (1.04–3.54); 0.04
Tolvaptan-treated series censored	−2.41 (−3.45, −1.37); <0.001	3.61 (1.59–8.19); 0.002
Never treated with tolvaptan	−2.90 (−4.52, −1.27); 0.001	3.82 (1.28–11.43); 0.02
Lifetime episode count, per category (trend)	−0.65 (−1.17, −0.13); 0.01	1.51 (1.14–2.01); 0.004

CI, confidence interval; eGFR, estimated glomerular filtration rate; ESKD, end-stage kidney disease; htTKV, height-adjusted total kidney volume; HR, hazard ratio; TKV, total kidney volume; UACR, urinary albumin-to-creatinine ratio.

Each cell: effect (95% CI); *P*. β in ml/min/1.73 m²/year (eGFR-slope model) unless the row denotes a per-category trend; HR, hazard ratio for the composite of ESKD or eGFR halving. All models are adjusted for baseline eGFR, age and sex; each row adds the stated covariate or restriction. The volume-adjusted estimate within the same subsample without the volume term was unchanged (β −2.07), confirming a covariate rather than a sample effect. TKV growth remained non-significant in every model (all *P *> .35). Full estimates for any and microscopic hematuria and additional scenarios are in Supplementary Tables S2–S5.

Subsequent disease-modifying therapy did not explain the findings. During follow-up 68 patients (39%) initiated tolvaptan and four sirolimus, all after the baseline period and balanced between groups. The macroscopic-hematuria association with eGFR decline persisted and increased when treated series were censored at initiation [−2.41 ml/min/1.73 m²/year (95% CI, −3.45 to −1.37); *P *< .001] and among never-treated patients [−2.90 (−4.52 to −1.27); *P *= .001; *n* = 88], versus −2.08 in the full cohort; the composite association strengthened correspondingly [adjusted HR 3.61 (1.59–8.19), *P *= .002 with censoring; 3.82 (1.28–11.43), *P *= .02 never-treated]. Modeling tolvaptan as a time-dependent covariate, macroscopic hematuria [HR 1.99 (1.09–3.63); *P *= 0.03] and time-varying tolvaptan exposure [HR 2.02 (1.04–3.89); *P *= .04] were each independently associated with the composite end point. A hematuria-by-tolvaptan interaction was significant (macroscopic: ordinary least-squares *P *= .03; Cox *P *= .009), in the direction of attenuation under treatment—consistent with a therapy-independent signal that is dampened, not produced, by tolvaptan.

Outcomes worsened with a greater burden of hematuria. For macroscopic hematuria modeled as lifetime episode count (0, 1, ≥2), there was a graded association with eGFR decline (trend *P *= .01) and with the composite end point [HR 1.51 per category (1.14–2.01); trend *P *= .004], the most consistent dose–response signal observed. Microscopic hematuria showed a significant episode-count trend for eGFR decline [*P *= .03; strengthened to *P *= .02 when infection-associated episodes were excluded, with a single-episode effect of −1.47 ml/min/1.73 m²/year (−2.62 to −0.31), *P *= .01] but only a borderline composite trend. Continuous intensity ratios were sensitive to their denominator, were not robust, and are not reported further.

Restricting TKV-growth estimation to determinations spanning at least one year, or more stringently to at least three determinations over one year, left the null association unchanged [macroscopic hematuria +0.59%/year (95% CI, −0.75 to 1.94), *P *= .39; and +0.61%/year (−0.69 to 1.90), *P *= .36, respectively; all exposures *P *> .35], indicating that the absence of a volume-growth association is not an artefact of short or noisy measurement intervals.

## DISCUSSION

In this long-term analysis of a well-characterized ADPKD cohort, hematuria documented early in the disease course independently predicted accelerated loss of kidney function over up to two decades of follow-up. Patients with baseline hematuria lost eGFR roughly 1–1.5 ml/min/1.73 m²/year faster after accounting for baseline kidney function, age and sex, and reached the composite end point of ESKD or eGFR halving earlier (15-year event-free survival 33% vs. 58%); the eGFR-slope association persisted after additional adjustment for baseline kidney volume and albuminuria. The association was most consistent for macroscopic hematuria but was also evident for microscopic hematuria, particularly when infection-associated episodes were excluded. Importantly, hematuria predicted functional decline but not the rate of kidney volume growth, indicating that its prognostic value reflects a functionally aggressive course rather than a simply larger or faster-growing kidney.

The dissociation between functional and volumetric progression is the central and most novel observation. TKV is the best-validated structural predictor of ADPKD progression [6] and forms the basis of the Mayo imaging classification, [7, 8, 22] yet it captures only part of the prognostic picture. That baseline hematuria adds information about eGFR decline without tracking TKV growth suggests it marks processes—cyst hemorrhage, vascular fragility, microvascular or tubulointerstitial injury—that translate into nephron loss disproportionate to cyst burden. Hematuria may thus serve as an inexpensive, routinely available functional risk marker that complements, rather than duplicates, volumetric staging. Consistent with a functional rather than volume-mediated marker, the association persisted after adjustment for baseline kidney volume, so hematuria is not merely a proxy for greater cyst burden; and the predominantly isomorphic urinary erythrocytes observed here point to a non-glomerular, cyst-associated or vascular source rather than glomerular injury. Whether hematuria is itself a cause of nephron loss or a surrogate of a more aggressive intrinsic course cannot be resolved by observational data; its dose-dependence and independence from kidney volume are compatible with either interpretation and support its use as a prognostic marker regardless of causal direction.

These considerations raise the possibility that baseline macroscopic hematuria functions primarily as a marker of a more aggressive underlying renal phenotype rather than as an independent mediator of nephron injury. Because bleeding in this cohort was predominantly non-glomerular, a direct mechanistic link between an individual hemorrhagic event and subsequent eGFR loss is difficult to posit; macroscopic hematuria may instead flag structurally more advanced or vascularly more fragile cystic disease. A parallel exists in IgA nephropathy, where a single baseline hematuria assessment carries limited prognostic weight, whereas the persistence and magnitude of hematuria during follow-up track more closely with outcome. Against a purely single-snapshot interpretation, our dose-response findings—cumulative macroscopic hematuria burden predicting eGFR decline (trend *P *= .01) and the composite end point (HR 1.51 per category; trend *P *= .004)—indicate a graded association more consistent with a phenotype-linked signal than with an artefact of one baseline observation. Mechanistic studies correlating hematuria with imaging, vascular and molecular markers will ultimately be required before it can be regarded as a therapeutic target rather than a prognostic signal.

Our findings extend earlier observations linking gross hematuria to adverse outcomes in ADPKD. Gabow and colleagues reported decades ago that episodes of gross hematuria were associated with larger kidneys and worse renal function [12] and identified hematuria among the clinical features marking a more severe phenotype [13]. Those analyses were largely cross-sectional or based on shorter follow-up. The present study advances this literature by using prospectively collected, serially measured eGFR over up to 20 years; quantifying progression as individual rates of decline with mixed-effects modeling; and linking a hard composite kidney end point to hematuria documented years earlier. The signal was consistent across continuous (eGFR slope) and time-to-event (renal survival) outcomes and across unadjusted, adjusted and mixed-model analyses, supporting its robustness.

The observation that excluding infection-associated episodes strengthened the association merits emphasis. Hematuria in ADPKD has heterogeneous causes—cyst rupture, nephrolithiasis [23], urinary tract infection—not all of which carry the same prognostic weight [11]. Infection-related hematuria is often transient, and its inclusion appears to dilute the signal of intrinsic disease activity. This argues for careful hematuria definitions in prognostic models and may explain inconsistent prior associations.

Macroscopic hematuria was a stronger and more consistent predictor than microscopic hematuria across end points. This gradient is biologically plausible: visible bleeding reflects more substantial cyst-wall disruption and vascular involvement than microscopic erythrocyturia. Nonetheless, microscopic hematuria remained independently associated with eGFR decline in the mixed-effects model, indicating that even subclinical erythrocyturia carries prognostic information and could be captured through routine urinalysis without additional cost [24]. In ordinary least-squares slope analysis the microscopic-hematuria estimate was of borderline significance (*P *= .07) and its prognostic signal was carried mainly by the mixed-effects model and by non–infection-associated episodes, whereas macroscopic hematuria was the more robust marker across all analyses. The prognostic signal was moreover independent of baseline albuminuria, an established predictor of progression in ADPKD [25].

Hypertension developed in the large majority of patients during follow-up, including all patients with baseline hematuria who were normotensive at baseline. Although point estimates were directionally consistent with an earlier onset of hypertension in the hematuria group, the near-universal penetrance of hypertension in ADPKD limited its usefulness as a discriminating end point in this cohort.

Strengths include a well-phenotyped cohort with standardized baseline urinary sediment microscopy, near-complete linkage (99%) to a longitudinal registry, densely sampled creatinine and imaging data, and follow-up long enough to observe definitive kidney end points. Our findings should be interpreted in the context of the study design. First, by design hematuria was ascertained at baseline, as our aim was to test whether an early, routinely available finding predicts long-term outcome; time-updated sediment status was not required for this question. Any reference patient developing hematuria later would, if anything, bias the association toward the null, so the estimates are conservative. Second, patients with hematuria had shorter observed follow-up, raising the possibility of informative censoring; however, a landmark analysis restricted to the first 8 years—when censoring is minimal—showed an association at least as strong as in the full follow-up. Mixed-effects models using all measurements, a hard composite end point and a joint longitudinal–survival model further mitigate this concern; the joint model confirmed the differential eGFR decline and indicated that the effect of macroscopic hematuria on ESKD is mediated through the eGFR trajectory. Third, eGFR decline was modeled as linear; a natural cubic spline did not improve fit over the observed period, although later nonlinear acceleration cannot be excluded [22]. Fourth, this is an observational analysis; despite multivariable adjustment, residual confounding cannot be excluded. Although the number of ESKD events analyzed alone was modest, the primary composite end point captured 64 events, and effect sizes were large and statistically significant. Although 39% of patients initiated tolvaptan during follow-up, sensitivity analyses censoring or excluding treated patients confirmed—and tended to strengthen—the associations. Genotype was not available; although it influences prognosis, its correlation with phenotype is imperfect, and its incremental value beyond the validated predictors we accounted for—baseline eGFR and, in sensitivity analyses, TKV—is likely limited. Concurrent urine microbiology was not systematically available, so urinary tract infection could be addressed through clinical flagging and sensitivity analyses rather than culture confirmation, and cross-sectional imaging was not separately adjudicated for cyst complications. Finally, the single-ancestry, single-registry cohort may limit generalizability.

In conclusion, baseline hematuria—especially macroscopic and non–infection-associated microscopic hematuria—independently predicts accelerated functional decline and worse long-term renal survival. Because this prognostic signal is functional rather than volume-driven, this simple, inexpensive and routinely obtained finding may help identify patients at higher risk of progression and complement established imaging-based risk markers [10].

## Supplementary Material

sfag300_Supplemental_File

## Data Availability

Owing to the small, single-center cohort and the consequent risk of patient re-identification, individual-level data are not deposited in a public repository. De-identified data and analysis code (Python and R) are available from the corresponding author on reasonable request, subject to cantonal ethics approval and Suisse ADPKD registry governance.

## References

[1] Cornec-Le Gall E, Alam A, Perrone RD. Autosomal dominant polycystic kidney disease. Lancet. 2019;393:919–35. 10.1016/S0140-6736(18)32782-X 30819518

[2] Cornec-Le Gall E, Audrézet MP, Chen JM et al. Type of PKD1 mutation influences renal outcome in ADPKD. J Am Soc Nephrol. 2013;24:1006–13. 10.1681/ASN.2012070650 23431072 PMC3665389

[3] Hateboer N, van Dijk MA, Bogdanova N et al. Comparison of phenotypes of polycystic kidney disease types 1 and 2. Lancet. 1999;353:103–7. 10.1016/S0140-6736(98)03495-3 10023895

[4] Torres VE, Chapman AB, Devuyst O et al. Tolvaptan in patients with autosomal dominant polycystic kidney disease. N Engl J Med. 2012;367:2407–18. 10.1056/NEJMoa1205511 23121377 PMC3760207

[5] Torres VE, Chapman AB, Devuyst O et al. Tolvaptan in later-stage autosomal dominant polycystic kidney disease. N Engl J Med. 2017;377:1930–42. 10.1056/NEJMoa1710030 29105594

[6] Grantham JJ, Chapman AB, Torres VE. Volume progression in autosomal dominant polycystic kidney disease: the major factor determining clinical outcomes. Clin J Am Soc Nephrol. 2006;1:148–57. 10.2215/CJN.00330705 17699202

[7] Irazabal MV, Rangel LJ, Bergstralh EJ et al. Imaging classification of autosomal dominant polycystic kidney disease: a simple model for selecting patients for clinical trials. J Am Soc Nephrol. 2015;26:160–72. 10.1681/ASN.2013101138 24904092 PMC4279733

[8] Chapman AB, Bost JE, Torres VE et al. Kidney volume and functional outcomes in autosomal dominant polycystic kidney disease. Clin J Am Soc Nephrol. 2012;7:479–86. 10.2215/CJN.09500911 22344503 PMC3302672

[9] Cornec-Le Gall E, Audrézet MP, Rousseau A et al. The PROPKD score: a new algorithm to predict renal survival in autosomal dominant polycystic kidney disease. J Am Soc Nephrol. 2016;27:942–51. 10.1681/ASN.2015010016 26150605 PMC4769200

[10] Kidney Disease: Improving Global Outcomes (KDIGO) ADPKD Work Group . KDIGO 2025 clinical practice guideline for the evaluation, management, and treatment of autosomal dominant polycystic kidney disease (ADPKD). Kidney Int. 2025;107:S1–S239. 39848759 10.1016/j.kint.2024.07.009

[11] Dedi R, Bhandari S, Turney JH et al. Causes of haematuria in adult polycystic kidney disease. BMJ. 2001;323:386–7. 10.1136/bmj.323.7309.386 11509433 PMC1120981

[12] Gabow PA, Duley I, Johnson AM. Clinical profiles of gross hematuria in autosomal dominant polycystic kidney disease. Am J Kidney Dis. 1992;20:140–3. 10.1016/S0272-6386(12)80541-5 1496966

[13] Johnson AM, Gabow PA. Identification of patients with autosomal dominant polycystic kidney disease at highest risk for end-stage renal disease. J Am Soc Nephrol. 1997;8:1560–7. 10.1681/ASN.V8101560 9335384

[14] Cohen RA, Brown RS. Microscopic hematuria. N Engl J Med. 2003;348:2330–8. 10.1056/NEJMcp012694 12788998

[15] Ravine D, Sheffield LJ, Danks DM et al. Evaluation of ultrasonographic diagnostic criteria for autosomal dominant polycystic kidney disease 1. Lancet. 1994;343:824–7. 10.1016/S0140-6736(94)92026-5 7908078

[16] Pei Y, Obaji J, Dupuis A et al. Unified criteria for ultrasonographic diagnosis of ADPKD. J Am Soc Nephrol. 2009;20:205–12. 10.1681/ASN.2008050507 18945943 PMC2615723

[17] Kouri T, Fogazzi G, Gant V et al. European urinalysis guidelines. Scand J Clin Lab Invest. 2000;60:1–96. 10.1080/00365513.2000.12056993 12647764

[18] Fairley KF, Birch DF. Haematuria: a simple method for identifying glomerular bleeding. Kidney Int. 1982;21:105–8. 10.1038/ki.1982.16 7077941

[19] Inker LA, Eneanya ND, Coresh J et al. New creatinine- and cystatin C-based equations to estimate GFR without race. N Engl J Med. 2021;385:1737–49. 10.1056/NEJMoa2102953 34554658 PMC8822996

[20] Kistler AD, Poster D, Krauer F et al. Increases in kidney volume in autosomal dominant polycystic kidney disease can be detected within 6 months. Kidney Int. 2009;75:235–41. 10.1038/ki.2008.558 18971924

[21] Rizopoulos D . JM: an R package for the joint modelling of longitudinal and time-to-event data. J Stat Soft. 2010;35:1–33. 10.18637/jss.v035.i09

[22] Yu ASL, Shen C, Landsittel DP et al. Long-term trajectory of kidney function in autosomal-dominant polycystic kidney disease. Kidney Int. 2019;95:1253–61. 10.1016/j.kint.2018.12.023 30922668 PMC6478515

[23] Nishiura JL, Neves R, Eloi SRM et al. Evaluation of nephrolithiasis in autosomal dominant polycystic kidney disease patients. Clin J Am Soc Nephrol. 2009;4:838–44. 10.2215/CJN.03100608 19339428 PMC2666433

[24] Vivante A, Afek A, Frenkel-Nir Y et al. Persistent asymptomatic isolated microscopic hematuria in Israeli adolescents and young adults and risk for end-stage renal disease. JAMA. 2011;306:729–36. 10.1001/jama.2011.1141 21846854

[25] Chapman AB, Johnson AM, Gabow PA et al. Overt proteinuria and microalbuminuria in autosomal dominant polycystic kidney disease. J Am Soc Nephrol. 1994;5:1349–54. 10.1681/ASN.V561349 7894001

