# Supplementary material for: Baseline hematuria in autosomal dominant polycystic kidney disease
Source: Clin Kidney J. 2026 Aug 31;19(9):sfag300. doi: 10.1093/ckj/sfag300 (PMC13569864; doi:10.1093/ckj/sfag300)
Supplement: Supplementary file 1 [file sfag300_supplemental_file.docx]

**Supplementary Tables**

Baseline hematuria and long-term kidney outcomes in autosomal dominant polycystic kidney disease.

**Supplementary Table S1.** Association of microscopic hematuria with eGFR slope and the composite kidney end point across alternative definitions. The eGFR slope β (ml/min/1.73 m²/year) and composite-endpoint hazard ratios are adjusted for baseline eGFR, age and sex.

| **Microhematuria definition** | **n exposed** | **eGFR slope β (95% CI); P** | **Composite HR (95% CI); P** |
| --- | --- | --- | --- |
| All sediment assessments | 35 | −0.92 (−1.90, 0.07); 0.07 | 1.80 (1.00–3.21); 0.05 |
| Regular visits | 33 | −0.96 (−1.96, 0.05); 0.06 | 1.53 (0.84–2.81); 0.17 |
| Regular, excluding infection | 29 | −1.38 (−2.42, −0.33); 0.01 | 1.51 (0.81–2.80); 0.20 |
| Clean (excl. infection & menstruation) | 27 | −1.03 (−2.13, 0.06); 0.06 | 1.51 (0.81–2.80); 0.20 |

*CI, confidence interval; eGFR, estimated glomerular filtration rate; HR, hazard ratio.*

**Supplementary Table S2.** Baseline-TKV, height-adjusted TKV (htTKV) and albuminuria (UACR)-adjusted associations of any, macroscopic and microscopic baseline hematuria with eGFR slope and the composite kidney end point. The reference (REF) model is adjusted for baseline eGFR, age and sex; subsequent rows add the stated covariate. β in ml/min/1.73 m²/year (hematuria versus none).

| **Exposure** | **Model** | **eGFR slope β (95% CI); P** | **Composite HR (95% CI); P** |
| --- | --- | --- | --- |
| Any | REF (eGFR, age, sex) | −1.44 (−2.34, −0.54); 0.002 | 1.58 (0.92–2.71); 0.10 |
|  | + TKV | −1.22 (−2.08, −0.37); 0.005 | 1.80 (1.04–3.11); 0.036 |
|  | + htTKV | −1.21 (−2.06, −0.35); 0.006 | 1.77 (1.02–3.06); 0.041 |
|  | + UACR | −1.32 (−2.23, −0.40); 0.005 | 1.32 (0.76–2.29); 0.32 |
|  | + UACR + TKV | −1.24 (−2.11, −0.37); 0.005 | 1.46 (0.83–2.57); 0.18 |
| Macroscopic | REF (eGFR, age, sex) | −2.08 (−3.12, −1.04); <0.001 | 1.97 (1.08–3.58); 0.03 |
|  | + TKV | −1.68 (−2.69, −0.66); 0.001 | 2.18 (1.19–4.00); 0.01 |
|  | + htTKV | −1.65 (−2.66, −0.64); 0.002 | 2.16 (1.18–3.96); 0.01 |
|  | + UACR | −1.96 (−3.02, −0.91); <0.001 | 1.92 (1.04–3.54); 0.04 |
|  | + UACR + TKV | −1.68 (−2.70, −0.66); 0.001 | 2.01 (1.08–3.72); 0.03 |
| Microscopic | REF (eGFR, age, sex) | −1.03 (−2.13, 0.06); 0.065 | 1.51 (0.81–2.80); 0.20 |
|  | + TKV | −1.01 (−2.03, 0.02); 0.055 | 1.68 (0.89–3.15); 0.11 |
|  | + htTKV | −0.99 (−2.02, 0.03); 0.057 | 1.68 (0.89–3.15); 0.11 |
|  | + UACR | −0.86 (−1.97, 0.25); 0.130 | 1.07 (0.56–2.04); 0.83 |
|  | + UACR + TKV | −1.03 (−2.09, 0.03); 0.056 | 1.23 (0.64–2.37); 0.53 |

*eGFR-slope set n=156 (155 with a volume term); composite set n=166 (164 with a volume term); UACR available in 173/174 linked patients. Reference estimates recomputed within each adjusted subsample were unchanged (e.g. macroscopic −2.07 to −2.08), indicating a covariate rather than a sample effect. CI, confidence interval; HR, hazard ratio; TKV, total kidney volume; htTKV, height-adjusted TKV; UACR, urinary albumin-to-creatinine ratio.*

**Supplementary Table S3.** Tolvaptan sensitivity analyses for eGFR slope and the composite kidney end point, by hematuria type. All models adjusted for baseline eGFR, age and sex. β in ml/min/1.73 m²/year (hematuria versus none).

| **Exposure** | **Scenario** | **eGFR slope β (95% CI); P** | **Composite HR (95% CI); P** |
| --- | --- | --- | --- |
| Any | Main (full cohort) | −1.44 (−2.34, −0.54); 0.002 | 1.58 (0.92–2.71); 0.10 |
|  | Treated series censored | −1.56 (−2.46, −0.65); <0.001 | 2.08 (0.92–4.70); 0.077 |
|  | Never treated with tolvaptan | −2.09 (−3.63, −0.55); 0.008 | 2.43 (0.69–8.56); 0.17 |
| Macroscopic | Main (full cohort) | −2.08 (−3.12, −1.04); <0.001 | 1.97 (1.08–3.58); 0.03 |
|  | Treated series censored | −2.41 (−3.45, −1.37); <0.001 | 3.61 (1.59–8.19); 0.002 |
|  | Never treated with tolvaptan | −2.90 (−4.52, −1.27); <0.001 | 3.82 (1.28–11.43); 0.02 |
| Microscopic | Main (full cohort) | −1.03 (−2.13, 0.06); 0.065 | 1.51 (0.81–2.80); 0.20 |
|  | Treated series censored | −0.93 (−2.04, 0.19); 0.102 | 1.51 (0.60–3.81); 0.39 |
|  | Never treated with tolvaptan | −1.34 (−3.22, 0.55); 0.164 | 1.87 (0.51–6.83); 0.35 |

*Full cohort: eGFR-slope n=156, composite n=166 (64 events); never-treated subset: eGFR-slope n=88, composite n=98 (17 events); censored composite 28 events. Time-dependent model (tolvaptan as a start–stop covariate): macroscopic hematuria HR 1.99 (1.09–3.63), P=0.03, and time-varying tolvaptan HR 2.02 (1.04–3.89), P=0.04, each independently associated with the composite end point. Hematuria-by-tolvaptan interaction (macroscopic): ordinary least-squares P=0.03; Cox P=0.009, in the direction of attenuation under treatment.*

**Supplementary Table S4.** Dose–response analyses: hematuria episode count (0, 1, ≥2) versus eGFR slope and the composite kidney end point, with a linear trend test. All models adjusted for baseline eGFR, age and sex. β per category in ml/min/1.73 m²/year.

| **Exposure (episode count)** | **Cell sizes 0/1/≥2** | **eGFR slope, β per category (95% CI); P(trend)** | **Composite HR per category (95% CI); P(trend)** |
| --- | --- | --- | --- |
| Macroscopic, lifetime | 119/12/25 | −0.65 (−1.17, −0.13); 0.014 | 1.51 (1.14–2.01); 0.004 |
| Macroscopic, baseline window | 128/14/14 | −0.88 (−1.50, −0.25); 0.006 | 1.32 (0.94–1.85); 0.105 |
| Microscopic, all episodes | 125/22/9 | −0.81 (−1.52, −0.10); 0.026 | 1.43 (0.99–2.06); 0.058 |
| Microscopic, infection-excluded | 130/20/6 | −0.93 (−1.74, −0.13); 0.023 | 1.24 (0.83–1.85); 0.304 |

*Cell sizes shown for the eGFR-slope set. Selected category contrasts: macroscopic lifetime 1 versus 0 β −1.56 (−3.01, −0.11), P=0.036; ≥2 versus 0 β −1.15 (−2.21, −0.09), P=0.034; composite 1 versus 0 HR 3.90 (1.75–8.67), P=0.001. Microscopic infection-excluded, 1 versus 0 β −1.47 (−2.62, −0.31), P=0.013. ≥2 categories are small (n=6–28) and are reported as trends. Continuous intensity ratios (episodes per visit or per year) were denominator-dependent and not robust.*

**Supplementary Table S5.** Total kidney volume growth (%/year) by baseline hematuria status under alternative minimum measurement intervals. All models adjusted for baseline eGFR, age and sex; β = hematuria versus none.

| **Minimum interval** | **Any β (95% CI); P** | **Macroscopic β (95% CI); P** | **Microscopic β (95% CI); P** |
| --- | --- | --- | --- |
| ≥0.5 year, ≥2 determinations (primary; n=154) | +0.40 (−0.73, 1.52); 0.49 | +0.61 (−0.73, 1.95); 0.37 | −0.24 (−1.56, 1.08); 0.72 |
| ≥1 year, ≥2 determinations (n=153) | +0.47 (−0.66, 1.60); 0.41 | +0.59 (−0.75, 1.94); 0.39 | −0.14 (−1.49, 1.20); 0.83 |
| ≥3 determinations over ≥1 year (n=148) | +0.47 (−0.63, 1.56); 0.40 | +0.61 (−0.69, 1.90); 0.36 | −0.15 (−1.44, 1.14); 0.82 |

*The absence of an association between hematuria and TKV growth is not explained by short or noisy measurement intervals: all exposures remained non-significant (P>0.35) under stricter interval requirements.*
